# Involvement of dopamine D3 receptor and dopamine transporter in methamphetamine‐induced behavioral sensitization in tree shrews

**DOI:** 10.1002/brb3.1533

**Published:** 2020-01-14

**Authors:** Jian Huang, Genmeng Yang, Zhen Li, Chi‐Kwan Leung, Wenguang Wang, Yuanyuan Li, Liu Liu, Baoyu Shen, Cuihua He, Yongwang He, Xiaofeng Zeng, Juan Li

**Affiliations:** ^1^ School of Forensic Medicine Kunming Medical University Kunming China; ^2^ School of Biomedical Sciences the Chinese University of Hong Kong Hong Kong China; ^3^ CUHK‐SDU Joint Laboratory of Reproductive Genetics School of Biomedical Sciences the Chinese University of Hong Kong Hong Kong China; ^4^ Center of Tree Shrew Germplasm Resources Institute of Medical Biology Yunnan Key Laboratory of Vaccine Research and Development on Severe Infectious Diseases Yunnan Innovation Team of Standardization and Application Research in Tree Shrew the Chinese Academy of Medical Science and Peking Union Medical College Kunming China; ^5^ School of Basic Medicine Kunming Medical University Kunming China

**Keywords:** behavioral sensitization, dopamine D3 receptor, dopamine transporter, METH, methamphetamine, tree shrews

## Abstract

**Introduction:**

This study aims to establish a methamphetamine (METH)‐induced behavioral sensitization model using tree shrews, as well as to measure the protein expression of the dopamine D3 receptor (D3R) and dopamine transporter (DAT).

**Methods:**

Forty tree shrews were equally and randomly divided into four experimental groups: those administered with 1, 2, and 4 mg/kg METH and a control group (treated with an equal amount of normal saline). Each experimental group was repeatedly exposed to METH for nine consecutive days to induce the development of behavioral sensitization, followed by four days of withdrawal (without the METH treatment) to induce the transfer of behavioral sensitization, then given 0.5 mg/kg of METH to undergo the expression of behavioral sensitization. Altered locomotor and stereotypic behaviors were measured daily via open‐field experiments during the development and expression stages, and weight changes were also recorded. Then, the Western blot method was used to detect the expression levels of D3R and DAT in three brain regions: the nucleus accumbens, prefrontal cortex, and dorsal striatum 24 hr after the last behavioral test.

**Results:**

METH administration augmented motor‐stimulant responses and stereotypic behaviors in all experimental groups, and stereotypic behaviors intensified more in the groups treated with 2 and 4 mg/kg METH. Motion distance, speed, and trajectory were significantly elevated in all experimental, however, METH at 4 mg/kg induced more stereotypic behaviors, decreasing these locomotor activities as compared with the 2 mg/kg METH group. 2 and 4 mg/kg METH significantly upregulated and downregulated D3R and DAT expression levels, respectively, in three brain regions, and these changes are more pronounced in 2 mg/kg METH.

**Conclusions:**

These results indicated that this animal model may be used to study the neurobiological mechanisms that underly the development and expression of behavioral sensitization to METH. Deregulated D3R and DAT expression may be involved in the METH‐induced behavioral sensitization.

## Graphical abstract

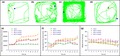

## 1 INTRODUCTION

Methamphetamine (METH), a white and odorless crystalline powder commonly known as “crystal METH,” is a highly addictive synthetic psychostimulant. It is a principal amphetamine‐type stimulant (ATS), the abuse of which has infiltrated mainstream culture across the Asia Pacific region. METH is characterized by its excitability of the central nervous system (CNS), strong drug dependence, high relapse rate, easy access to raw materials, simple synthesis process and low production cost, the combination of which have made METH abuse an ever‐growing phenomenon. By the end of 2018 in China, 1.35 million out of 2.4 million registered drug abusers were METH abusers, posing a significant socioeconomic burden (Annual report, 2017: The situation of the drug problem in China, 2018).

Chronic METH consumption elicits compulsive drug craving and frequent relapse as well as other pathological behaviors (Miner, Phillips, & Janowsky, 2019). METH enters dopaminergic presynaptic terminals mediated by the dopamine transporter (DAT) to promote dopamine (DA) release into the cytosol, redistribute dopamine stores and elevate cytosolic DA abundance partly via the vesicular monoamine transporter‐2 (VMAT2) (Friedman, Castaneda, & Hodge, 1998). METH was shown to inhibit mitochondrial enzyme monoamine oxidase (MAO), abrogating the cellular metabolism of DA, resulting in behavioral deficits, memory loss, and neurotoxic effects in the CNS (Zhou et al., 2019).

Behavioral sensitization refers to the enhancement of behavioral responses to repeated and intermittent drug exposure, including augmented autonomic activity and stereotypic behavior (Steketee & Kalivas, 2011). Drug‐induced behaviorally sensitized animal models are used routinely to define the cellular and molecular mechanisms underlying drug addiction and associated psychomotor behavioral alternations (Collins et al., 2011), and relevant tools have been developed to this effect (Kai, Nishizawa, Tsutsui, Ueda, & Kobayashi, 2015; Mohd‐Yusof et al., 2016). Further, pharmaceutical interventions can be introduced during the development of behavioral sensitization to evaluate the therapeutic effect on behavioral sensitization and reinstatement (Song et al., 2014; Sun et al., 2016; Zhao et al., 2014). The principal preclinical animal models of behavioral sensitization were rats (Mohd‐Yusof et al., 2016; Song et al., 2014; Zhao et al., 2014) and mice (Kai et al., 2015; Sun et al., 2016). Considering the significant differences in neuroanatomic structures and the functional divergence of the nervous systems of rodents and humans, however, these rodent‐based models may contribute limited understandings of addictive drugs' neurotoxicity and addictive behaviors in humans.

A number of studies have shown that the tree shrew (*Tupaia belangeri*) has a much closer phylogenetic affinity to primates than rodents do (Chen et al., 2019; Fan et al., 2013; Ma et al., 2013). Compared with rodents, the tree shrew has a more developed brain at both the neuroanatomical and the neurophysiological levels, higher homology of neuropeptidomics, similar drug target protein sequences and a higher resemblance to humans in terms of the expression profiles of genes associated with numerous neuropsychiatric disorders (Chen et al., 2019; Fan et al., 2013; Ma et al., 2013). However, the tree shrew has yet to be adopted as a preclinical animal model for studying METH‐induced behavioral sensitization.

The mechanism of METH addiction has been studied intensively in the field of biomedicine. The neural circuits associated with METH addiction are very complex, involving multiple brain regions (Kai et al., 2015; Xu et al., 2019) and a multitude of neurotransmitters (Jiang et al., 2018; Jing, Liu, Zhang, & Liang, 2018) and protein mediators (Lee, Kim, Kim, Lee, & Jang, 2018; Mohd‐Yusof et al., 2016; Sun et al., 2016; Zhao et al., 2014). The incentive‐sensitization theory of addiction is one of the most widely accepted classical theories used to explain METH‐induced behavioral sensitization. Addictive drugs, as opposed to natural rewards (e.g., food, water), act on the brain's reward system to induce the excessive attribution of incentive salience to drug‐associated stimuli, producing compulsive motivation and the ingraining of drug‐taking habits. METH promotes the synaptic release of DA and inhibits the DAT reuptake of DA, thereby activating DA receptors and dopaminergic signaling in the brain's reward pathway to elicit reward‐motivated behavior (Sun et al., 2016; Volz, Hanson, & Fleckenstein, 2007). Studies have shown that the D3R plays an important role in METH addiction, and it has been shown to relate closely to METH‐induced hyperactivity and behavioral sensitization in animal models (Collins et al., 2011; Jiang et al., 2018; Song et al., 2014; Sun et al., 2016). To continue these findings, the present study examines the protein expression of D3R and DAT in METH‐induced behavioral sensitization in tree shrews.

In this study, tree shrews are exposed to different doses of METH by intraperitoneal injection. METH‐induced stereotypic activities and psychomotor behavioral responses such as locomotor distance, speed, and moving track are then examined using open‐field tests. The biological relevance of D3R and DAT in this METH‐induced behavioral sensitization model is then characterized. Establishing a METH‐induced behavioral sensitization model using tree shrews could lead to the delineation of the molecular and cellular mechanisms underlying psychostimulant effects and drug dependence. Thus, this study could provide a scientific basis for future mechanistic studies on drug addiction and psychostimulant‐induced behaviors, with the potential to inform the development of pharmacological interventions against drug dependence and relapse.

## 2 METHODS

### 2.1 Animals

Male tree shrews (120–160 g, 1‐year‐old) were supplied by the Center of Tree Shrew Germplasm Resources, the Institute of Medical Biology, the Chinese Academy of Medical Science and Peking Union Medical College (Kunming, China). They were housed in a standard 12 hr:12 hr light/dark cycle at a room temperature of 23 ± 2℃, with access to food and water ad libitum. All experiments were approved by the Institutional Ethics Committee of Kunming Medical University and were performed in accordance with the ethical standards described in the NIH guidelines.

### 2.2 Drugs and antibodies

L‐methamphetamine hydrochloride (C_10_H_15_N⋅HCl) was purchased from the National Institutes for Food and Drug Control (Cat #: 171212‐200603, Beijing, China). The METH was dissolved in phosphate‐buffered saline (PBS) and administered to the tree shrews via intraperitoneal injection. The following antibodies were used: anti‐rabbit dopamine receptor D3 (Cat #: ab42114, Abcam, 1:1,000), anti‐rabbit dopamine transporter (Cat #: AB1591P, EMD Millipore, 1:1,000), anti‐rabbit β‐Actin (Cat #: 21,338, 1:1,000, Signalway Antibody, USA), and anti‐mouse/rabbit IgG and horseradish peroxidase‐linked secondary antibody (Cat #: L3012, 1:5,000, Signalway Antibody, USA).

### 2.3 Behavior sensitization experiment

During the development (d4–d12) and expression (d17) stages of METH‐induced behavioral sensitization, open‐field tests were used to evaluate the behavior sensitization of the tree shrews. The chosen apparatus (Shanghai XinRuan Information Technology Co, Ltd) consisted of an XR‐XZ301 (100*100*120 cm) chamber and a SONY Super HAD CCD camera, and the SuperMaze^+^ behavioral trajectory analysis system recorded all locomotor activity.

**Figure 1:**
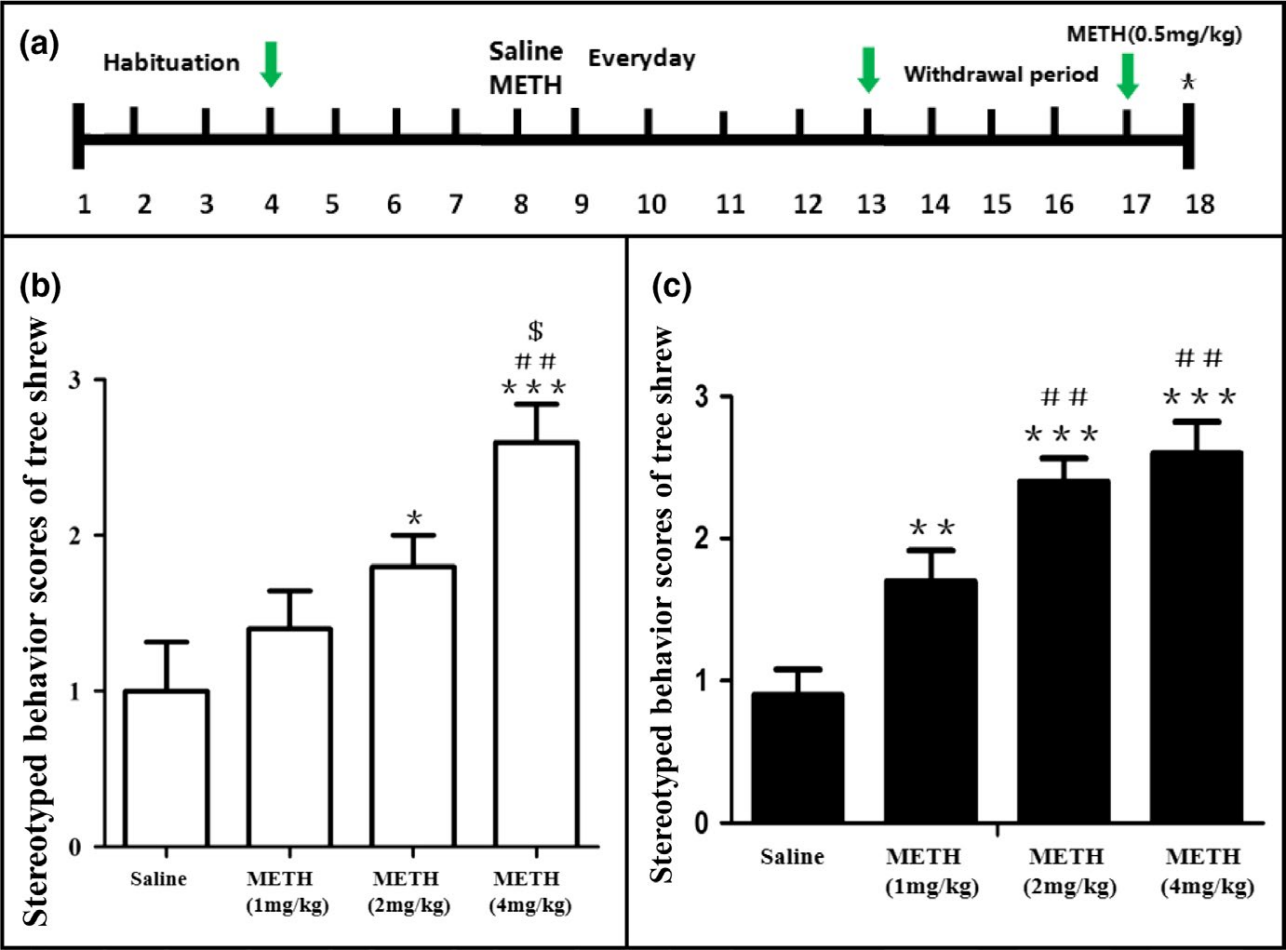
(a) Experimental protocol for METH and saline treatments. (b) Effects of different doses of METH on stereotypic behavior scores of behavioral sensitization in tree shrews on the last day (d12) of the development period of behavioral sensitization and (c) the expression period of behavioral sensitization (d17). *p < .05, **p < .01, and ***p < .001 compared to the control group. ^##^ *p* < .01 compared to METH (1 mg/kg). ^$^ *p* < .05 compared to METH (2 mg/kg). Data were presented as mean ± *SEM*. *N* = 10 per group

Forty male tree shrews were equally randomized into four experimental groups: those administered with 1, 2, and 4 mg/kg METH and a control group (given the equivalent amount of normal saline). As shown in Figure 1a, the animals were habituated in the chamber for 1 hr daily for 3 days (d1–3) before the drug treatment. In the development period of behavioral sensitization (d4–12), the animals in each group received METH or saline per the set dosage at a regular time per day and were immediately placed in the chamber for testing. The animals then experienced a 4‐day withdrawal period (d13–16) without the METH or saline treatment before being administered with 0.5 mg/kg METH or equivalent amount of normal saline the next day. Changes of behavior, movement distance, average speed, and motion trajectory were recorded for 1 hr daily during the development and expression periods, and stereotypic behavior scores and weight changes were also recorded.

### 2.4 Stereotyped behavior scores

After watching the video recordings made during the open‐field tests and referring to the GHF‐Dodd scoring method (Xie, Zhou, Zhang, Chen, & Wang, 2018), stereotypic behavior scores were made as follows (Table 1).

**Table 1:** The scoring system of the stereotyped behavior

Score	Behavior description
0	Stationary, little or no movement
1	Normal movement accompanied by repeated exploration in situ or no fixed direction of repeated exploration
2	Ran fast around the open field, circled, climbed, and jumped repeatedly
3	Repetitive movements of the head or tail, either up or down, directed toward one wall or corner of the chamber, experienced repeated convulsions and hiccups or sustained an arched position

### 2.5 Western blot

All tree shrews were euthanized using 40 mg/kg of 1% pentobarbital sodium salt (Sigma) via intraperitoneal anesthesia 24 hr after the last behavioral test, after which the PFC, DS, and NAc were harvested from each specimen and frozen in liquid nitrogen for use in subsequent experiments. Brain tissues (20 mg) from each encephalic region were homogenized in 150 μl of protein extraction buffer (Beyotime, Shanghai, China) containing protease and phosphatase inhibitors, then centrifuged at 14,000 *g* for 15 min at 4℃. The supernatant was collected, and the proteins were measured using the Bradford Protein Assay kit (Beyotime). After the protein sample loading buffer was added, samples were boiled at 99℃ for 10 min. The samples were then separated by 8% SDS‐PAGE and transferred to 0.45 µm polyvinylidene difluoride (PVDF) membranes (Millipore). The membranes were blocked in 5% nonfat dry milk (diluted in the Tris‐buffered saline with 0.1% Tween 20 (TBST)) for 1 hr at room temperature, then incubated in appropriate primary antibodies (1:1,000 dilution with 5% defatted milk) overnight at 4℃. Next, the membranes were washed three times for 10 min each with TBST and incubated with the secondary antibody (1:5,000 dilution with 5% defatted milk) for 1 hr at room temperature. Finally, the membranes were detected using an enhanced chemiluminescent Plus Detection kit (Millipore, USA) and visualized using a Bio‐Rad Imaging system (Bio‐Rad). This experiment was repeated in triplicate, and representative Western blot images were presented.

### 2.6 Data analysis

Statistical analyses were performed using SPSS 21.0 (IBM SPSS) and GraphPad Prism 7.00 (GraphPad Software). All data were represented as the mean ± *SEM* of 10 animals for behavioral assays and three independent biological replicates for the Western blot. Stereotyped behavior scores, motion trajectory, and Western blot data were analyzed using a one‐way analysis of variance (ANOVA) and analyzed post‐hoc using LSD test. Movement distance and average speed were analyzed using a two‐factor (dose x time) ANOVA with repeated measures (time) and analyzed post‐hoc using LSD test. A paired *T*‐test was used in the intragroup comparison to analyze the weight changes between d12/d17 and d4. *p*‐values of <.05 were considered statistically significant.

## 3 RESULTS

### 3.1 METH‐augmented stereotyped behavior

During the development (d4–d12) and expression (d17) stages of METH‐induced behavioral sensitization (Figure 1a), spontaneous stereotypic activity started within the first few minutes in all experimental groups postinjection. The behaviorally sensitized animals ran back and forth rapidly in the open field and exhibited forward circling, repetitive vertical jumping and backward somersaulting. After approximately 15 min, stereotypic behaviors were observed in all experimental groups, the animals exhibited irritability, forward exploration, repetitive head shaking and tail curling to a semicurled shape, as well as constant shaking, rapid crying, burping, and increased defecation. The saline‐paired control group did not display aberrant stereotypy at any stage. As shown in Figure 1b,c, the intensity of stereotypy increased with higher METH dosages. On the last day (d12) of the development period of behavioral sensitization, 2 and 4 mg/kg METH significantly increased stereotyped behavior scores compared to the control group (*F*_3, 36_ = 16.154, *p < .05 and ***p < .001), and METH at 4 mg/kg exhibited higher scores than the 1 and 2 mg/kg group (*F*_3, 36_ = 16.154, ^##^ *p* < .01 and ^$^ *p* < .05). On the expression period of behavioral sensitization (d17), the scores were all significantly increased compared to the control group at different METH doses (*F*_3, 36_ = 15.478, **p < .01 and ***p < .001), and 2 and 4 mg/kg METH exhibited higher scores than the 1 mg/kg group (*F*_3, 36_ = 15.478, ^##^ *p* < .01). These results suggest that METH promotes stereotypic behavioral sensitization in the tree shrews.

### 3.2 METH‐induced biphasic effect in locomotion

**Figure 2:**
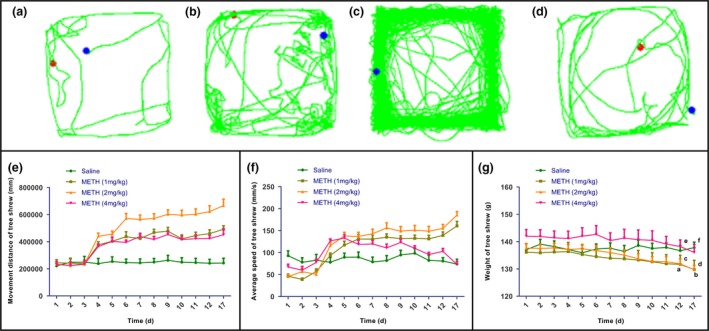
Effects of different doses of METH on motion trajectory, movement distance, average speed, and body weight of the tree shrews. The enclosed photograph illustrates the change of motion trajectory diagram for the tree shrews during the expression period of behavioral sensitization (d17) for control group (a), METH at 1 mg/kg (b), 2 mg/kg (c), and 4 mg/kg (d). (e) Effects of different doses of METH on movement distance of the tree shrews. (f) Effects of different doses of METH on average speed of the tree shrews. (g) Effects of different doses of METH on weight of the tree shrew (a = *p*_1 mg/kg_ < .001 on d12; b = *p*_1 mg/kg_ < .001 on d17; c = *p*_2 mg/kg_ < .001 on d12; d = *p*_2 mg/kg_ < .001 on d17; e = *p*_4 mg/kg_ < .01 on d12; f = *p*_4 mg/kg_ < .01 on d17). Data were presented as mean ± *SEM*. *N* = 10 per group

Next, METH‐induced behavioral sensitization on the locomotor response of the tree shrews was examined using open‐field tests. Figure 2a‐d shows the change of the motion trajectory diagram for the tree shrews during the expression period of behavioral sensitization (d17). Hyperlocomotion was observed in all experimental groups postinjection. One‐way ANOVA revealed the enhancement of locomotor response in all experimental groups compared to the control group (*F*_3, 36_ = 20.022, *p* < .001), and METH at 2 mg/kg resulted in a maximal locomotor response compared to the 1 and 4 mg/kg group (*F*_3, 36_ = 20.022, *p* < .01 and *p* < .001). These results suggest that the METH induced a biphasic effect in locomotion, with an initial peak of locomotor response at 2 mg/kg followed by suppression at 4 mg/kg.

Then, we tested the effects of repeated METH administration at different doses on movement distance and average speed using two‐factor ANOVA with repeated measures. As shown in Figure 2e, there was a statistically significant of METH doses main effect (*F*_3, 36_ = 31.256, *p* < .001), time main effect (*F*_5.151, 185.438_ = 9.237, *p* < .001), and interaction of these two factors (*F*_5.151, 185.438_ = 2.264, *p* = .005) on movement distance. The simple main effects analysis showed that the movement distance of the tree shrews increased significantly after the first METH administration in all experimental groups (*p* < .001, as compared to the saline‐paired controls). The behaviorally sensitized animals' movement distances during the development (d4 ‐ d12) and expression stage (d17) were all significantly increased compared to the control group at different METH doses (*p* < .001). METH at 2 mg/kg significantly increased movement distance compared to the 1 mg/kg group (*p* < .001). However, METH at 4 mg/kg failed to increase movement distance further compared to the 2 mg/kg group (*p* < .001).

As shown in Figure 2f, there was a statistically significant of METH doses main effect (*F*_3, 36_ = 16.343, *p* < .001), time main effect (*F*_9, 324_ = 3.471, *p* < .001), and interaction of these two factors (*F*_27, 324_ = 6.531, *p* < .001) on average speed. The simple main effects analysis showed that the average speed of the tree shrews increased significantly after the first administration of METH in all dosage groups (*p* < .001, as compared to the saline‐paired controls). Next, the average speeds of the behaviorally sensitized animals during the development (d4–d12) and expression stage (d17) were all significantly increased compared to the control group at different METH doses (*p* < .001 for the 1 and 2 mg/kg groups; *p* = .008 for the 4 mg/kg group compared to the control group). Similar to the effect of METH on movement distance, however, METH at 4 mg/kg decreased average speed compared to the 2 mg/kg group (*p* < .001).

These cumulative results indicate that METH doses significantly augment the movement distances and average speeds of tree shrews during the development and expression stages of behavioral sensitization. METH at 2 mg/kg produces the most significant increases in movement distance and average speed. Tree shrews mainly exhibit stereotypic behaviors at 4 mg/kg METH.

### 3.3 METH‐induced weight loss in behaviorally sensitized animals

The tree shrews were weighed once a day before the drug was given; average weights exhibited a decreasing trend in all experimental groups. As shown in Figure 2g, the tree shrews' weights decreased significantly on d12 and d17 across all groups compared to their weights before the administration of METH (*p*_1 mg/kg_ < .001 on d12; *p*_1 mg/kg_ < .001 on d17; *p*_2 mg/kg_ < .001 on d12; *p*_2 mg/kg_ < .001 on d17; *p*_4 mg/kg_ < .01 on d12; *p*_4 mg/kg_ < .01 on d17). The control group did not display such weight loss at any stage. These results suggest that METH at any examined dose causes a significant reduction in body weight.

### 3.4 METH‐regulated endogenous expression of D3R and DAT in behaviorally sensitized animals

**Figure 3:**
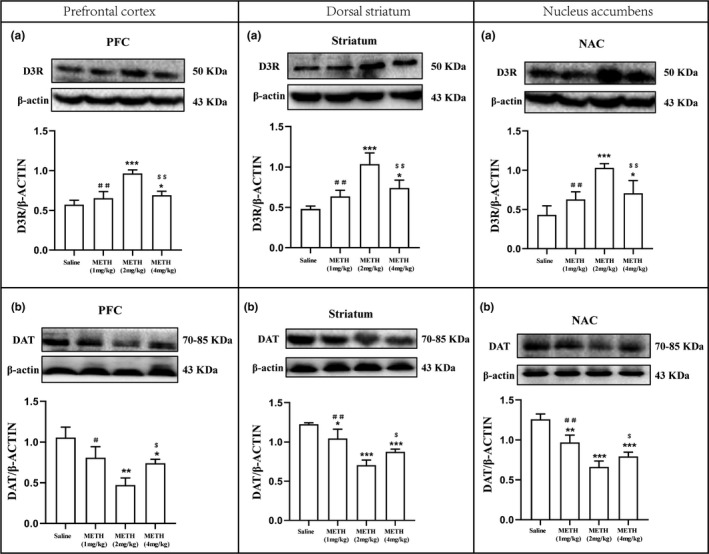
Regulation of different doses of METH to the expressions of D3R and DAT in the prefrontal cortex (PFC) (left panel a and b), dorsal striatum (DS) (middle panel a and b), and nucleus accumbens (NAc) (right panel a and b) per the METH‐induced behavioral sensitization model of the tree shrews. β‐actin‐normalized protein levels of D3R and DAT were determined using Western blot. Representative blot images are shown. *p < .05, **p < .01, ***p < .001 compared to the saline‐paired control group. ^＃^ *p* < .05, ^＃＃^ *p* < .01 compared to the 2 mg/kg METH group. ^＄^ *p* < .05, ^＄＄^ *p* < .01 compared to the 2 mg/kg METH group. Data were presented as mean ± *SEM*. *N* = 3 per group of the behaviorally sensitized tree shrews

Prior studies indicate that both D3R and DAT are of biological significance in the neuronal circuitry underlying METH‐induced behavioral sensitization and reinstatement. In this investigation, the protein expression levels of D3R and DAT in the PFC, DS, and NAc of behaviorally sensitized tree shrews exposed to METH were examined. As shown in Figure 3a, 2 and 4 mg/kg METH significantly upregulated D3R expression levels in PFC (*F*_3, 8_ = 24.240, *p < .05 and ***p < .001), DS (*F*_3, 8_ = 18.679, *p < .05 and ***p < .001), and NAc (*F*_3, 8_ = 14.383, *p < .05 and ***p < .001) compared to the control group, and in these three brain regions, these changes are more pronounced in 2 mg/kg METH compared with METH at 1 and 4 mg/kg (^##^ *p* < .01 and ^$$^ *p* < .01). Figure 3b shows that 1, 2, and 4 mg/kg METH significantly downregulated DAT expression levels in PFC (*F*_3, 8_ = 9.628, *p < .05 and **p < .01), DS (*F*_3, 8_ = 30.173, *p < .05 and ***p < .001), and NAc (*F*_3, 8_ = 37.651, **p < .01 and ***p < .001) compared to the control group, and in these three brain regions, these changes are more pronounced in 2 mg/kg METH compared with METH at 1 and 4 mg/kg (^#^ *p* < .05, ^##^ *p* < .01 and ^$^ *p* < .05). These results indicate that METH regulates the expression of D3R and DAT in the three key domains of forebrain terminals for drug addiction‐associated behavioral anomalies.

## 4 DISCUSSION

In this study, a METH‐induced behavioral sensitization model was established using tree shrews, and the expression levels of D3R and DAT in METH‐induced behavioral sensitization were examined.

The spontaneous stereotypy of all METH‐treated experimental groups grew significantly with each increase in METH dosage (Figure 1). Though any of the three tested dosage of METH can induce profound behavioral sensitization, METH at 4 mg/kg was observed to reduce locomotor ability significantly; mainly, stereotypic behaviors manifested, suggesting that sensitization to high doses of METH can escalate to the manifestation of augmented stereotypic activities that compete with locomotion. After the first administration of METH, the motor distance and average motor speed of the tree shrews in all drug groups increased significantly compared to those in the control group (Figure 2). METH at 1 and 2 mg/kg increased the motor distance and speed, but changes in rigid behavior were slight. With 4 mg/kg METH, the tree shrews' motor distance and speed were inhibited, and rigid behavior was obvious.

The average body weight of the tree shrews in each experimental group showed a significant downward trend as the experiment progressed, presumably associated with augmented locomotor and stereotypic activities, resulting in enhanced energy expenditure (Figure 2). This indicates that METH can induce obvious behavioral sensitization in the tree shrews, which is consistent with the results of previous animal behavior studies in terms of dosage and behavioral characteristics (Wearne, Parker, Franklin, Goodchild, & Cornish, 2017; Zhu et al., 2012). On the other note, prior studies indicated that METH use suppresses appetite, directly disrupts the gastrointestinal system and leads to malnutrition and weight loss (Fantegrossi et al., 2008). METH consumption has also been exploited for weight control (Bruening, Perez La Mar, & Ohrt, 2018). It is therefore speculated that these toxic effects induced by METH may also occur in the tree shrews. These two factors could be the reasons why METH induced the loss of body weight in the tree shrews.

The present study focused on D3R (Chen, Song, Yang, Wu, & Li, 2014; Le Foll et al., 2014), which is a DR subtype closely related to METH addiction. Prior studies have shown that D3R is highly expressed in PFC, NAc, and DS (Ares‐Santos, Granado, & Moratalla, 2013; Wang et al., 2018). Thus, changes in D3R and DAT protein expression in the PFC, NAc, and DS of the midbrain were examined via a behavioral sensitization model of METH‐exposed tree shrews. Our results indicate that 2 and 4 mg/kg of METH significantly reduced the expression levels of DAT in the three brain regions but significantly increased the expression levels of D3R (Figure 3). METH at 2 mg/kg induces the most significant change in the protein expression levels of both D3R and DAT in the three brain regions. The changes in the protein expression of D3R and DAT in PFC, DS, and NAc seem to correlate with the behavioral alternations in terms of the locomotor and stereotypy in METH‐induced behavioral sensitization model of tree shrews. We showed that METH administration heightened locomotor responses and stereotypic behaviors in all experimental groups. METH at 2 mg/kg, maximally promotes and inhibits the protein expression of D3R and DAT, respectively, elicits the most significant increases in movement distance and average speed and moderately augments stereotypic behaviors. METH at 1 mg/kg, weakly upregulates and downregulates D3R and DAT expression levels, respectively, produces the minimal elevation on the locomotor and stereotypy. Tree shrews mainly exhibit stereotypic behaviors at 4 mg/kg METH, which produces the strongest stereotypy and moderately augments locomotor behavior. The results suggest that METH can regulate the endogenous expressions of D3R and DAT, which are of biological significance in the METH‐induced behavioral sensitization of tree shrews.

Studies have shown that D3R knockout mice exhibit a lack of behavioral sensitization after acute and chronic morphine administration, suggesting that morphine‐induced behavioral sensitization in mice may require D3R (Li et al., 2010). D3R can regulate morphine‐induced behavioral sensitization in mice by downregulating N‐methyl‐D‐aspartate receptor subunit (NR2B) expression in NAc (Liu et al., 2014). Likewise, D3R is a pivotal mediator in the regulation of METH‐induced acute motor and behavioral sensitization in mice (Liu et al., 2014). This study shows for the first time that D3R may also contribute to METH‐induced behavioral sensitization in tree shrews.

Studies have shown that METH can not only lead to the depletion of DA but also cause the modification of DAT structures by forming a polymer complex with it. Moreover, there is a negative correlation between the formation of DAT complexes induced by METH and DAT activity (Hadlock et al., 2009). The present study shows that METH can significantly reduce the DAT expression in three brain regions of tree shrews, suggesting that METH may form a complex with DAT and reduce its expression and activity in different brain regions. This is consistent with previous studies demonstrating that drug abuse with substances such as METH, amphetamine, and cocaine reduces DAT levels. METH abuse can induce the release of large amounts of DA, inhibit DA reuptake and eventually induce the release of large amounts of DA, thereby reducing the expression of DAT.

Thus, D3R and DAT may play very important roles in METH‐induced behavioral sensitization in tree shrews.

## 5 CONCLUSIONS

We established and characterized a METH‐induced behavioral sensitization model using tree shrews, and METH regulates the expression of D3R and DAT in PFC, DS, and NAc for METH‐associated behavioral sensitization. This newly established behavioral sensitization model for tree shrews will be useful to those studying the neural basis of addiction. Such investigations could lead to the development of pharmacotherapies to treat drug dependence and psychostimulant‐induced behaviors.

## CONFLICT OF INTEREST

The authors declare no conflicts of interest.

## Data Availability

The data that support the findings of this study are available from the corresponding author upon reasonable request.
